# Nocturnal Ballistic Bouts in ADCY5-Related Movement Disorder

**DOI:** 10.7759/cureus.101726

**Published:** 2026-01-17

**Authors:** Bhadra Sajeev Nair, Boby Varkey Maramattom

**Affiliations:** 1 Department of Medicine, Malankara Orthodox Syrian Church Medical College Hospital, Kolenchery, IND; 2 Department of Neurology, Aster Medcity, Kochi, IND

**Keywords:** adcy5-related movement disorder, adenylyl cyclase, ballistic bouts, dyskynesia, dystonia

## Abstract

Adenylyl cyclase 5 (ADCY5)-related movement disorder (ADCY5-RMD) is a rare genetic hyperkinetic movement disorder caused by pathogenic variants in the ADCY5 gene, characterized by childhood-onset chorea, dystonia, myoclonus, and distinctive paroxysmal exacerbations, often with nocturnal worsening. The disorder exhibits marked phenotypic variability, minimal disease progression, and frequently normal neuroimaging, leading to frequent misdiagnosis as dyskinetic cerebral palsy or epilepsy.

We report the case of a 60-year-old woman with childhood-onset generalized dystonia, prominent oromandibular and lingual involvement, asymmetric distal choreoathetosis, and recurrent nocturnal ballistic bouts beginning at age 13. Episodes occurred predominantly during sleep-wake transitions and were exacerbated by stress. Neurological examination and brain magnetic resonance imaging (MRI) were unremarkable. Whole-exome sequencing (WES) identified a heterozygous de novo splice-site mutation in ADCY5 (c.2088+1G>A), classified as pathogenic. Symptomatic treatment with trihexyphenidyl, clonazepam, and quetiapine led to a significant reduction in episode frequency and severity.

This case expands the phenotypic spectrum of ADCY5-RMD by demonstrating persistence of sleep-related paroxysmal dyskinesia into late adulthood and highlights the importance of considering ADCY5-RMD in long-standing childhood-onset hyperkinetic movement disorders.

## Introduction

Adenylyl cyclase 5 (ADCY5)-related movement disorder (ADCY5-RMD) is a rare hyperkinetic movement disorder caused by pathogenic variants in the ADCY5 gene, which encodes ADCY5. Onset is usually in infancy or adolescence, with episodic chorea, dystonia, myoclonus, especially facial myoclonus, and athetosis. Disease severity varies widely, even among individuals with the same mutation, and some carriers remain asymptomatic [1]. Associated symptoms include continuous dysarthria, episodic orofacial dystonia, and progressive axial hypotonia. A hallmark feature of ADCY5-RMD is the presence of paroxysmal exacerbations of hyperkinetic movements or ballistic bouts [2,3].

The ADCY5 gene encodes adenylyl cyclase 5, a membrane-bound enzyme that catalyzes the conversion of adenosine triphosphate to cyclic adenosine monophosphate (cAMP), a key second messenger involved in neuronal signaling within basal ganglia motor circuits [3].

We report the case of a 60-year-old female patient with ADCY5-RMD who suffered from severe nocturnal ballistic bouts. 

ADCY5-RMD remains under-recognized, particularly in adults with childhood-onset hyperkinetic movement disorders. Most existing reports focus on pediatric presentations, and data on the persistence of characteristic nocturnal paroxysmal exacerbations into late adulthood are limited. Documenting such cases is important to expand the recognized clinical spectrum, improve diagnostic awareness, and emphasize the role of genetic testing in patients with long-standing, non-progressive hyperkinetic movement disorders that are frequently misdiagnosed [3,4].

## Case presentation

In June 2024, a 60-year-old female presented with a history of generalized dystonia, with prominent involvement of the oromandibular region. Lingual dystonia was also noted, accompanied by distal choreoathetotic movements. These involuntary, sustained, and repetitive movements were reported to have begun in childhood. The dyskinetic episodes were asymmetrical, with a greater severity noted in the left limbs compared to the right. Extraocular movements were normal, and a Kayser-Fleischer ring was absent. The events followed a consistent and predictable sequence, typically occurring during the transition from wakefulness to sleep. Exacerbations were reported during periods of physiological stress, such as febrile illness or emotional distress. Developmental history was significant for delayed motor milestones. The patient experienced her first episode at age 13, characterized by involuntary ballistic movements lasting approximately two to three minutes.

Cognitive function was preserved, with no history of intellectual disability, seizures, syncope, or altered consciousness. No autonomic dysfunction, psychiatric comorbidity, dysphagia, aspiration, or significant weight loss was reported. Cardiopulmonary disease was absent. Nocturnal ballistic bouts caused sleep disruption and occasional minor self-injury, without major trauma. Family history was negative for movement disorders or neurodegenerative disease.

Routine laboratory investigations were largely unremarkable. Brain magnetic resonance imaging (MRI) revealed no structural abnormalities. A genetic study comprising whole exome sequencing (WES) revealed a de novo splice site (C-2088 + 1 G>A mutation) in the ADCY5 gene. The patient was initiated on trihexyphenidyl 1 mg/day, clonazepam 1.5 mg/day, and quetiapine 25 mg/day. Following this, the frequency of nocturnal episodes decreased significantly, from five to six episodes per week to two to three episodes per fortnight. Using a patient-reported 10-point Visual Analog Scale (VAS) for episode severity, scores decreased from an estimated 8-9/10 prior to treatment to 4-5/10 after treatment initiation.

In addition to reduced episode frequency, the patient reported a subjective reduction in episode severity, describing the movements as “less violent” and of shorter duration. Prior to treatment, nocturnal episodes frequently resulted in sleep interruption and daytime fatigue; following treatment initiation, the patient reported improved sleep continuity and reduced fear of nocturnal attacks. Functionally, she was able to sleep independently without injury or need for supervision.

The patient was followed for a period of six months after initiation of therapy. Treatment was well tolerated, with no serious adverse effects reported. Mild daytime somnolence was noted during the initial weeks of clonazepam therapy, which improved without dose adjustment. No anticholinergic side effects, behavioral changes, or extrapyramidal symptoms were observed. There was no worsening of cognition, mood, or functional status during follow-up. The therapeutic benefit was sustained throughout the follow-up period, with continued reduction in the frequency and severity of nocturnal episodes and no emergence of new neurological symptoms.

## Discussion

ADCY5 is one of nine membrane-bound isoforms of adenylyl cyclase that catalyze the conversion of adenosine triphosphate (ATP) into pyrophosphate and cAMP, a critical second messenger involved in numerous intracellular signaling pathways. ADCY5 is predominantly expressed in the brain and cardiac tissue, with particularly high expression levels in the striatum, a key component of the basal ganglia implicated in motor control. This region-specific expression pattern underlies the association between ADCY5 dysfunction and movement disorders [3].

The pathogenic mechanisms of ADCY5-RMD are primarily linked to mutations affecting the enzyme’s structure and function. For example, splice-site mutations within one of the two cytoplasmic catalytic domains of the ADCY5 protein may result in aberrant mRNA splicing or degradation, leading to haploid insufficiency. However, the majority of identified ADCY5 mutations are gain-of-function variants, which enhance enzymatic activity and consequently elevate intracellular cAMP concentrations in neurons. This dysregulated cAMP signaling perturbs normal neuronal excitability and synaptic transmission, particularly within motor circuits of the brain, ultimately manifesting as involuntary hyperkinetic movements, including chorea, dystonia, and myoclonus [2,3].

**Figure 1:**
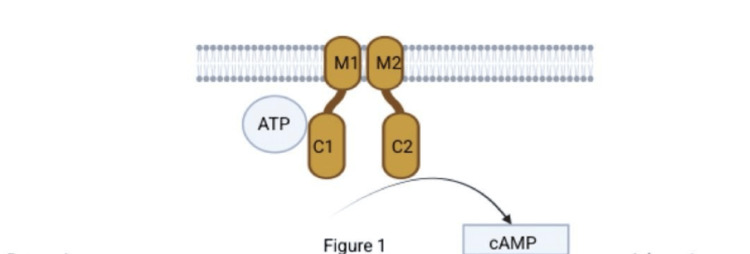
Structure of Adenylyl Cyclase 5 (ADCY5) ADCY5 is a membrane-bound enzyme composed of two transmembrane regions, M1 and M2, that anchor it within the cell membrane. Within the cytoplasm, ADCY5 contains two catalytic domains, C1 and C2, which interact to convert ATP into cyclic AMP (cAMP). This enzymatic reaction plays a pivotal role in intracellular signal transduction in neurons. Image credits: Created by the authors on BioRender software (BioRender, Toronto, Canada)

**Figure 2:**
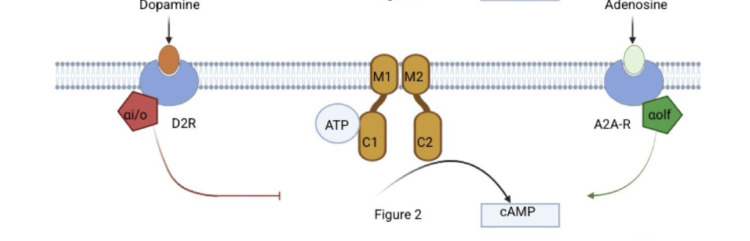
Normal Regulation of Adenylyl Cyclase 5 (ADCY5) in the Brain Neurons ADCY5 activity is tightly regulated by two G protein-coupled receptors: the adenosine A2A receptor and the dopamine D2 receptor. Activation of the A2A receptor by adenosine stimulates the G protein subunit Gαolf, which in turn activates ADCY5, resulting in increased cyclic AMP (cAMP) production. Conversely, activation of the D2 receptor by dopamine engages Gαi/o proteins, which inhibit ADCY5 and reduce cAMP levels. The dynamic interplay between these opposing signals maintains a physiological balance of cAMP signaling in neurons. Image credits: Created by authors on BioRender software (BioRender, Toronto, Canada)

**Figure 3:**
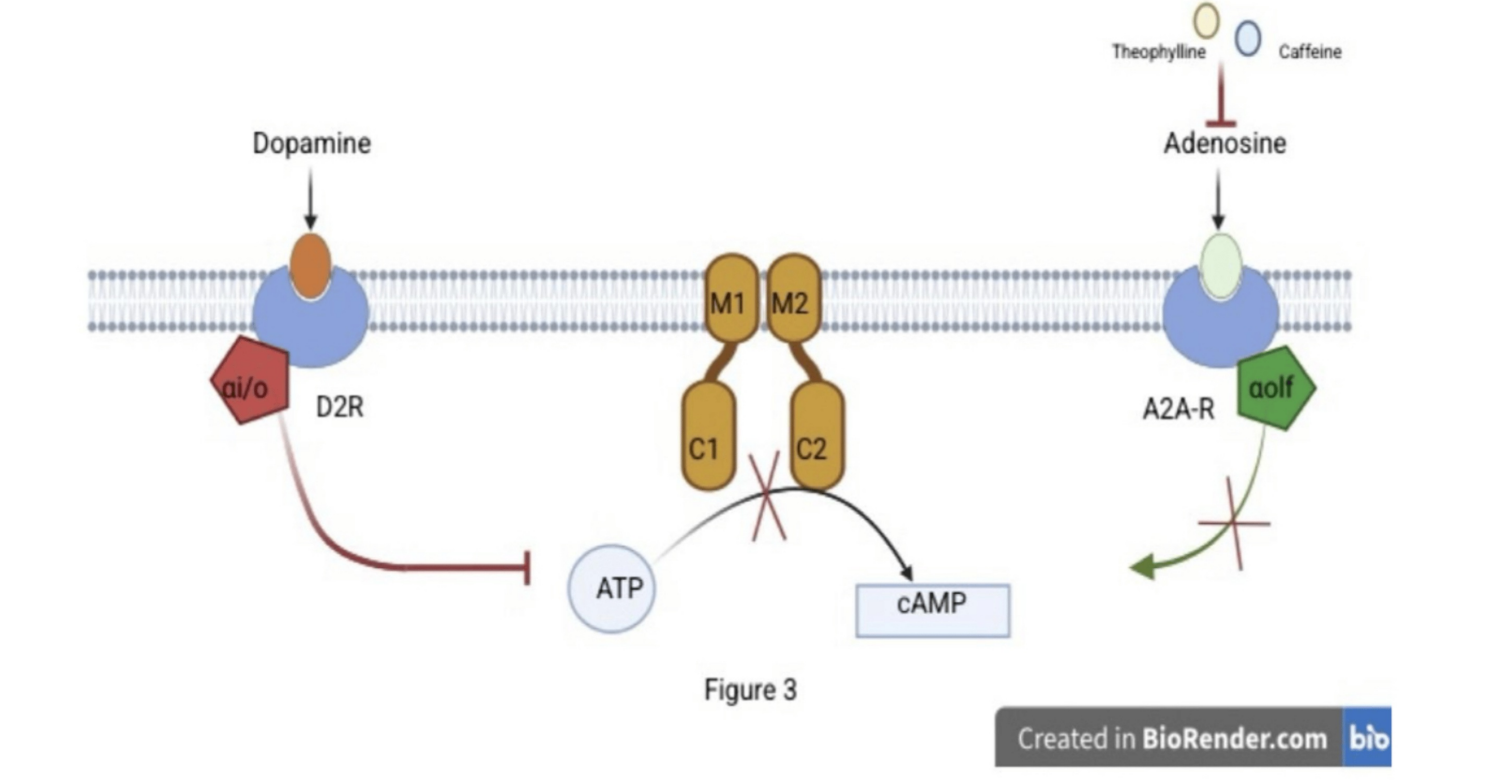
Pharmacological Inhibition of A2A Receptors Reduces Adenylyl Cyclase 5 (ADCY5) Activity A2A receptor antagonists, such as caffeine, theophylline, and istradefylline, block the stimulatory effects of adenosine on ADCY5. This prevents activation of Gαolf, leading to decreased stimulation of ADCY5 and reduced cyclic AMP (cAMP) synthesis. Meanwhile, the D2 receptor remains active and continues to suppress ADCY5 through Gαi/o signaling. The combined effect is a significant overall reduction in cAMP production within neuronal cells. Image credits: Created by authors on BioRender software (BioRender, Toronto, Canada)

ADCY5-RMD typically has an autosomal dominant inheritance pattern with variable expressivity and incomplete penetrance. De novo mutations and cases of somatic mosaicism have also been documented [4]. The underlying pathophysiology involves gain-of-function mutations in ADCY5, resulting in increased intracellular cAMP in striatal neurons (Figure 1). Adenosine A2A receptors (A2AR) stimulate ADCY5, so A2AR antagonists such as caffeine and theophylline can reduce cAMP levels and are proposed as first-line therapies (Figures 2-3).

WES identified a heterozygous de novo splice-site variant in the ADCY5 gene (c.2088+1G>A). Based on American College of Medical Genetics and Genomics (ACMG) criteria, including a predicted null effect on splicing (PVS1), de novo occurrence (PS2), and absence from population databases (PM2), the variant was classified as pathogenic. The mutation is predicted to disrupt normal RNA splicing, resulting in an abnormal or truncated ADCY5 protein. While splice-site variants may theoretically cause loss-of-function, pathogenic ADCY5 mutations are predominantly associated with functional dysregulation of cAMP signaling consistent with a gain-of-function disease mechanism [4].

Nocturnal ballistic bouts are especially problematic in ADCY5-RMD. Ballistic bouts are especially prominent during sleep or transitions between sleep and wakefulness, or during NREM-1 and REM sleep stages. Ballistic bouts are sudden, intense, and involuntary flinging or jerky movements involving the limbs and trunk and may last from seconds to up to 30 minutes, disrupting sleep and resulting in injury to self or the partner. Consciousness is preserved during these episodes, and there is no post-ictal state. Ballistic bouts resemble biballism and are more intense than the patient’s baseline dyskinesia. These episodes are a distinctive feature of ADCY5-RMD [5]. Ballistic bouts can also occur during wakefulness and may last minutes or hours, being triggered by drowsiness, awakenings, or other conditions, such as concurrent illnesses, emotions, anxiety, laughter, sneezing, and/or medications. Other genetic early-onset choreas such as NKX2.1, ADCY5, PDE10A, PDE2A, GNAO1, and OPA3 mutations are not associated with nocturnal paroxysmal dyskinesia or ballistic bouts [4,5].

The principal site of neuropathological involvement in ADCY5-RMD is the striatum, a key component of the basal ganglia that includes the caudate nucleus and putamen. ADCY5 is abundantly expressed in this region, highlighting its central role in the modulation of movement. Mutations in ADCY5 lead to aberrant intracellular signaling within striatal neurons, primarily through dysregulation of cAMP pathways. This functional disruption interferes with normal motor circuit activity, resulting in involuntary hyperkinetic movements that are characteristic of ADCY5-RMD [5,6].

ADCY5-RMD presents in early life with a combination of chorea, dystonia, and myoclonus. Nocturnal or sleep-related exacerbations are characteristic and often a diagnostic clue. The clinical features in this case, particularly the presence of ballistic nocturnal movements, oromandibular dystonia, developmental motor delay, and stress-related exacerbations, are strongly consistent with this disorder. The patient was found to harbor a heterozygous splice site mutation in the ADCY5 gene. The identified variant affects RNA splicing and is predicted to result in an aberrant or truncated adenylyl cyclase 5 protein [6]. Video-polysomnography is required to document the non-epileptic character of BB. Our patients had undergone a panoply of tests and were unwilling to undergo a PSG.

As ADCY5-RMD is a rare condition, it is misdiagnosed as drug-resistant epilepsy or other paroxysmal movement disorders. The diagnosis of ADCY5-RMD should be considered in patients with movement disorders, particularly when sleep-related episodes or nocturnal exacerbations are present [5]. 

In clinical practice, caffeine (300 mg/day) has shown benefit, leading to decreased frequency and intensity of paroxysms, improved sleep quality, and enhanced daytime functioning. Retrospective studies report that up to 87% of patients experience symptomatic improvement with caffeine. Theophylline and istradefylline have also demonstrated similar benefits. Some cases may, however, paradoxically worsen with caffeine [6].

Additional treatment options include benzodiazepines (e.g., clonazepam, clobazam), propranolol, acetazolamide, trihexyphenidyl, tetrabenazine, and levodopa, though responses are inconsistent. Of these, benzodiazepines appear most effective. Withdrawal of clonazepam may result in symptom exacerbation. For patients with drug-refractory symptoms, bilateral pallidal deep brain stimulation (DBS) has been used successfully, including in cases presenting with dyskinetic storms [6]. 

Bilateral pallidal DBS can reduce both continuous hyperkinetic movements and paroxysmal episodes in ADCY5-RMD, including improvements in facial dyskinesia, speech, and nocturnal exacerbations. However, axial dystonia is often less responsive, and overall functional gains are limited. Stopping stimulation leads to symptom worsening, regardless of treatment duration [5,6].

 Despite several therapeutic strategies, there is currently no disease-modifying treatment for ADCY5-RMD. Management remains symptomatic, with no established consensus guidelines or randomized controlled trials. Early identification and tailored therapy, particularly using A2AR antagonists, can significantly improve quality of life in affected individuals [6].

Symptomatic improvement in this patient was reflected by a reduction in patient-reported episode severity on a 10-point VAS, from 8-9/10 prior to treatment to 4-5/10 following therapy. This objective patient-reported outcome supports a clinically meaningful reduction in symptom burden in this case [7].

## Conclusions

This case illustrates that ADCY5-RMD can persist into late adulthood with prominent sleep-related paroxysmal exacerbations despite minimal disease progression. The presence of childhood-onset hyperkinetic movements, stress and sleep-associated worsening, and normal neuroimaging should prompt consideration of ADCY5-RMD even in older patients. Identification of a pathogenic ADCY5 splice-site variant confirmed the diagnosis and avoided further misclassification as epilepsy or other paroxysmal disorders. Symptomatic improvement with anticholinergic and benzodiazepine therapy highlights the potential for meaningful clinical benefit, even when diagnosis is delayed.

It should be noted that while the present patient demonstrated symptomatic improvement with anticholinergic and benzodiazepine therapy, other treatment strategies discussed, particularly A2AR antagonists and deep brain stimulation, are supported by prior literature and were not directly evaluated in this case.

This report is limited by its single-patient design, which restricts the generalizability of the clinical and therapeutic observations. Objective confirmation of the non-epileptic nature of the nocturnal ballistic bouts with PSG was not performed, as the patient declined further investigations.
